# Hepatitis C Virus Non-Structural Protein 5A (NS5A) Disrupts Mitochondrial Dynamics and Induces Mitophagy

**DOI:** 10.3390/cells8040290

**Published:** 2019-03-29

**Authors:** Alagie Jassey, Ching-Hsuan Liu, Chun A. Changou, Christopher D. Richardson, Hsue-Yin Hsu, Liang-Tzung Lin

**Affiliations:** 1 International Ph.D. Program in Medicine, College of Medicine, Taipei Medical University, Taipei 11031, Taiwan; alagie_jassey@yahoo.com; 2 Graduate Institute of Medical Sciences, College of Medicine, Taipei Medical University, Taipei 11031, Taiwan; julia.chliu@gmail.com; 3 Department of Microbiology & Immunology, Dalhousie University, Halifax, NS B3H 4R2, Canada; chris.richardson@dal.ca; 4 Ph.D. Program for Cancer Biology and Drug Discovery, College of Medical Science and Technology, Taipei Medical University, Taipei 11031, Taiwan; ptm@tmu.edu.tw; 5 Integrated Laboratory, Center of Translational Medicine, Taipei Medical University, Taipei 11031, Taiwan; 6 Core Facility, Taipei Medical University, Taipei 11031, Taiwan; 7 Department of Pediatrics and Canadian Center for Vaccinology, Izaak Walton Killam Health Centre, Halifax, NS B3H 4R2, Canada; 8 Department of Life Sciences, Tzu-Chi University, Hualien 970, Taiwan; hsueyin@mail.tcu.edu.tw; 9 Department of Microbiology and Immunology, School of Medicine, College of Medicine, Taipei Medical University, Taipei 11031, Taiwan

**Keywords:** HCV, NS5A, mitophagy, Parkin, mitochondrial dynamics

## Abstract

Mitophagy is a selective form of autophagy, targeting damaged mitochondria for lysosomal degradation. Although HCV infection has been shown to induce mitophagy, the precise underlying mechanism and the effector protein responsible remain unclear. Herein, we demonstrated that the HCV non-structural protein 5A (NS5A) plays a key role in regulating cellular mitophagy. Specifically, the expression of HCV NS5A in the hepatoma cells triggered hallmarks of mitophagy including mitochondrial fragmentation, loss of mitochondrial membrane potential, and Parkin translocation to the mitochondria. Furthermore, mitophagy induction through the expression of NS5A led to an increase in autophagic flux as demonstrated by an accumulation of LC3II in the presence of bafilomycin and a time-dependent decrease in p62 protein level. Intriguingly, the expression of NS5A concomitantly enhanced reactive oxygen species (ROS) production, and treatment with an antioxidant attenuated the NS5A-induced mitophagy event. These phenomena are similarly recapitulated in the NS5A-expressing HCV subgenomic replicon cells. Finally, we demonstrated that expression of HCV core, which has been documented to inhibit mitophagy, blocked the mitophagy induction both in cells harboring HCV replicating subgenomes or expressing NS5A alone. Our results, therefore, identified a new role for NS5A as an important regulator of HCV-induced mitophagy and have implications to broadening our understanding of the HCV-mitophagy interplay.

## 1. Introduction

Hepatitis C virus infects approximately 3% of the world’s population [1], predisposing the majority to end-stage liver diseases such as liver cirrhosis and hepatocellular carcinoma (HCC) [2]. The enveloped virus, against which no effective vaccine currently exists, belongs to the Flaviviridae family; it possesses a single-stranded RNA genome encoding a single polyprotein that is processed by host and viral proteases into structural (core and envelope glycoproteins E1 and E2) and non-structural (ion channel p7, NS2, NS3, NS4A, NS4B, NS5A, and NS5B) proteins [3].

The HCV NS5A is a 447 aa long, pleiotropic phosphoprotein with no known enzymatic activity. It exists in a basal phosphorylated form (56 kDa) or hyperphosphorylated form (58 kDa) and is an important regulator of the HCV life cycle [4,5]. NS5A is organized into three domains—domain I (aa 1–213), II (aa 250–342), and III (aa 355–447), which are separated by low complexity sequences 1 and 2, respectively [6]. Domain II contains a region called the interferon (IFN) sensitivity determining region (ISDR) in which mutations are assumed to be responsible for IFN resistance in certain hepatitis C patient populations [7]. NS5A has been shown to play an important role in the replication and assembly of the virus, interference of the host cell IFN response, and modulation of cellular signaling transduction [4]. It has been found to interact with numerous cellular pathways via its conserved polyprotein cluster [8], which matches the Src homology 3 binding motif of many signaling molecules [9]. Interestingly, NS5A has recently been reported to induce mitochondrial fragmentation [10], which is important for the induction of mitophagy.

Mitophagy is a specialized form of autophagy (or more specifically, macroautophagy) that delivers damaged mitochondria to the lysosomes for degradation via the double-membrane autophagosomes [11]. By removing damaged mitochondria, mitophagy plays an invaluable role in reducing cellular stress caused by the triggered oxidative burst and, in turn, helps maintain cellular homeostasis. In general, many steps involved in mitophagy overlap with non-selective autophagy, as they both involve the formation of an autophagosome that engulfs the cargo, and its maturation to an autophagolysosome for degradation. However, unlike non-specific autophagy involving bulk degradation of old or dysfunctional cytoplasmic contents, mitophagy requires additional molecular regulators for the selective degradation of the mitochondria. Key among these regulators include the E3 ubiquitin ligase, Parkin, predominantly localized in the cytoplasm [12], and the PTEN-induced putative kinase 1 (PINK1), a serine-threonine kinase [13]. In polarized mitochondria, PINK1 is exported to the mitochondria but its level is kept low by the presenilin-associated rhomboid-like protease (PARL) [14]. In depolarized mitochondria however, PINK1 accumulates on the outer mitochondria membrane and triggers the translocation of Parkin to the mitochondria. Parkin then ubiquitinates itself and the outer mitochondrial proteins, thereby priming the mitochondria for autophagic degradation [15].

HCV infection has been suggested to alter mitochondrial dynamics, promote Parkin translocation to the mitochondria, and subsequently induce mitophagy [16], but the precise underlying mechanism and the major effector protein responsible for the HCV-induced mitophagy remain elusive. Due to the pleiotropic nature of NS5A and its ability to modulate numerous cellular activities, in this study, we examined its potential involvement in the induction of mitophagy. Our results demonstrated that the HCV phosphoprotein NS5A can independently induce alterations in the mitochondrial dynamics that culminate in the induction of mitophagy, as evidenced by Parkin translocation and LC3II upregulation in the mitochondrial fraction of NS5A-expressing cells. Moreover, using the LC3 turnover and p62 degradation assays, we showed that overexpression of NS5A leads to complete autophagy (autophagic flux). Importantly, the presence of NS5A simultaneously triggered ROS production, inhibition of which with an antioxidant attenuated the NS5A-induced mitophagy. Furthermore, using western blotting and confocal microscopy analysis, we demonstrated that HCV subgenomic replicon cells efficiently induced mitophagy. Moreover, the expression of HCV core, which has been recently shown to physically interact with Parkin and prevent its translocation to the mitochondria, inhibited mitophagy in both cells harboring the HCV replicating subgenomes and expressing NS5A. Together, our results identified NS5A as a regulator of HCV-induced mitophagy with implications in advancing our knowledge of HCV-host cell interaction.

## 2. Materials and Methods

### 2.1. Chemicals and Reagents

Bafilomycin A1 (BAF) and dimethyl sulfoxide (DMSO) were purchased from Sigma-Aldrich (St. Louis, MO, USA). Carbonyl cyanide m-chlorophenyl hydrazone (CCCP) was purchased from Abcam (Cambridge, UK). Rapamycin, the inhibitor of the mammalian target of rapamycin (mTOR), was purchased from InvivoGen (San Diego, CA, USA). MitoTracker Deep Red, Dulbecco’s modified Eagle’s medium (DMEM), fetal bovine serum (FBS), gentamycin, and amphotericin B were purchased from Life Technologies (Carlsbad, CA, USA). Phosphate buffered saline (PBS) was purchased from Thermo Fisher Scientific (Waltham, MA, USA).

### 2.2. Cell Culture and Plasmids

The human hepatoma Huh-7 and Huh-7.5 cells were grown in DMEM supplemented with 10% FBS, 1% gentamycin, and 1% amphotericin B, and incubated at 37 °C in a 5% CO_2_ incubator. The HCV subgenomic replicon AB12-A2 cells are Huh-7 cells established with genotype 1b subgenomic HCV RNA [17] and were maintained in DMEM containing 1 mg/mL of G418 (InvivoGen). The plasmids pCMV-Tag1-NS5A (N-terminally FLAG-tagged NS5A; genotype 1b), CMV-FLAG-Core-R (pMO29), and pEGFP-LC3 were obtained from Addgene (Cambridge, MA, USA). The pDEST-mCherry-EGFP-LC3 plasmid [18] was a kind gift from Dr. Craig McCormick (Dalhousie University, Halifax, NS, Canada). For all transfection experiments, the plasmids were transfected into the cells using OMNIfect transfection reagent (Transomic Technologies; Huntsville, AL, USA). Mock controls are Huh-7.5 cells without transfection/treatment unless otherwise indicated. Huh-7.5 cells stably expressing NS5A were established by transfection of the pCMV-Tag1-NS5A into naïve Huh-7.5 cells followed by G418 (1 mg/mL) selection.

### 2.3. Fluorescence Microscopy

Huh-7.5 cells were seeded on glass slides overnight in a 24-well plate (6 × 10^4^ cells/well) and transfected with 2 μg of pCMV-Tag1-NS5A for 3 days. As positive control, naïve Huh-7.5 cells were treated with CCCP (10 μM) for 12 h prior to starting the staining procedures. The cells were subsequently pre-stained with MitoTracker Deep Red (100 nM) for 20 min in growth media, washed once with PBS, and fixed with 4% paraformaldehyde (PFA) for 30 min. Then, the cells were permeabilized for 30 min with 0.3% Triton X-100 (Sigma-Aldrich) at room temperature, blocked with 3% bovine serum albumin (BSA; Sigma-Aldrich), and incubated with the specific primary antibodies at 4 °C overnight: rabbit polyclonal anti-Parkin antibody (Abcam) at 1:400; mouse anti-Hepatitis C Virus, NS5A monoclonal antibody (Millipore; Billerica, MA, USA) at 1:250. Following additional PBS washes, the slides were probed with the respective secondary antibodies at 1:400 dilution: Alexa Fluor 488 goat anti-rabbit and Alexa Fluor 555 goat anti-mouse IgG (H + L) antibodies (Thermo Fisher Scientific). Images were taken with a TCS SP5 Confocal Spectral Microscope Imaging System (Leica; Wetzlar, Germany). For analyzing the effect of HCV core expression on the AB12-A2 HCV replicon-induced mitophagy, cells were seeded on glass slides in 24-well plates (6 × 10^4^ cells/well) and transfected with or without HCV core (1 μg) for 48 h before pre-staining with MitoTracker Deep Red, followed by immunostaining with anti-Parkin antibodies. For EGPF-LC3 and mCherry-EGFP-LC3 fluorescence microscopy, Huh-7.5 cells were seeded in 12-well plates (1 × 10^5^ cells/well) and transfected with the respective plasmids (pEGFP-LC3 and pDEST-mCherry-EGFP-LC3) alone at 1 μg each, or co-transfected with pCMV-Tag1-NS5A for 48 h before analysis. Rapamycin (100 nM) treatment was initiated at 44 h post-transfection and terminated at the 48 h end-point. For experiments with the pDEST-mCherry-EGFP-LC3 transfections, the mCherry-EGFP-LC3 groups with or with NS5A overexpression were further treated with 0.2 μM BAF for 12 h. Results were analyzed using a fluorescence microscope. Nuclei were demarcated with Hoechst stain (Sigma-Aldrich).

### 2.4. Mitochondria Isolation

The mitochondrial fractions were isolated using the Mitochondria Isolation Kit for Cultured Cells (Abcam) with some modifications. Cells were lifted with a cell lifter and centrifuged at 1000× *g* for 10 min at 4 °C. The cell pellets were resuspended to 5 mg/mL with reagent A, incubated on ice for 10 min, then sonicated and spun at 1000× *g* for 10 min. The supernatants were saved and the cell pellets were resuspended to the same concentration with reagent B, sonicated, and spun for 10 min at 4 °C. Finally, the two supernatants were thoroughly mixed and spun at 12,000× *g* for 15 min. The deposit (mitochondrial fraction) was resuspended with reagent C and kept at −80 °C until analysis.

### 2.5. Mitochondrial Membrane Potential Measurement

The mitochondrial membrane potential was measured with the JC10 Mitochondrial Membrane Potential Assay Kit (Abcam) using flow cytometry according to the manufacturer’s instructions. Huh-7.5 cells were seeded on a 6-well plate at a density of 5 × 10^5^ cells/well and transfected with pCMV-Tag1-NS5A for 3 days. The cells were subsequently washed with PBS, trypsinized, and resuspended with 500 μL of JC10 loading dye for 20 min incubation at room temperature. The fluorescent intensities for both J-aggregates (red) and monomeric forms (green) of JC10 were measured by standard flow cytometry and analyzed with the CellQuest software (Version 6.0, BD Biosciences; San Jose, CA, USA).

### 2.6. Western Blotting

Cells were lysed with RIPA buffer (Sigma-Aldrich) supplemented with cOmpleteTM Tablets Mini Protease Inhibitor Cocktail (ROCHE; Basel, Switzerland) and incubated on ice for 30 min, after which the lysates were clarified at 12,000 rpm for 30 min. The lysates were transferred into a new tube and the protein concentrations were determined using the Bio-Rad Protein Assay Kit II (Bio-Rad Laboratories; Hercules, CA, USA). The whole cell lysates were separated by SDS-PAGE and transferred to polyvinylidene difluoride (PVDF) membranes. The membranes were then probed with the following primary antibodies: rabbit anti-Parkin antibody (Abcam) at 1:2000; mouse anti-COX-4 (Santa Cruz Biotechnology; Santa Cruz, CA, USA) at 1:500, respectively; rabbit anti-LC3 antibody (Thermo Fisher Scientific) at 1:1000; rabbit anti-p62 antibody (GeneTex Inc.; Irvine, CA, USA) at 1:2000; and mouse anti-Hepatitis C Core antigen (C7-C50) (Thermo Fisher Scientific) at 1:500. The mouse monoclonal anti-NS5A (9E10) was a kind gift from Dr. Charles M. Rice of Rockefeller University (New York, NY, USA) and was used at 1:12500. The secondary antibodies used in the experiments included goat anti-rabbit IgG H&L HRP (Abcam) at 1:3000 and anti-mouse IgG HRP (Thermo Fisher Scientific) at 1:3000. The membranes were overlaid with ECL (Bio-Rad) and the images were taken with a ChemiDoc-ItTS2 imager (UVP; Upland, CA, USA). The relative signal intensity was quantified using ImageJ software (version 1.410) developed by W. Rasband (National Institutes of Health, Bethesda, MD, USA).

### 2.7. ROS Production and Scavenging Analysis

ROS production was assayed using 2′,7′-dichlorodihydrofluorescein diacetate (H_2_DCFDA; Sigma-Aldrich). Briefly, Huh-7.5, Huh-7.5/NS5A, or AB12-A2 cells were seeded in 6-well plates at 5 × 10^5^ cells/well. The following day, the respective cells were treated with or without 20 mM of the ROS scavenger N-acetyl-cysteine (NAC; Sigma-Aldrich) for 48 h, or induced with 1 mM hydrogen peroxide (H_2_O_2_) for 30 min. At 3 days post seeding, the cells were stained with 20 μM H_2_DCFDA for 30 min at 37 °C. Following washing with PBS, the cells were subsequently trypsinized, washed twice with PBS again, then resuspended in ice-cold PBS before flow cytometry analysis using the CellQuest software (BD Biosciences). For Western blot analysis of NAC treatment, Huh-7.5/NS5A cells were seeded in 10 cm dishes overnight before treatment with 20 mM NAC for 48 h. The mitochondrial fractions and the whole cell lysates from the 10 cm dishes were obtained as described earlier then subjected to Western blot analysis.

### 2.8. Statistical Analysis

GraphPad Prism software (Version 7.03, GraphPad Software; San Diego, CA, USA) was used for the statistical analysis. Values represent mean ± standard deviation (SD). At least three independent experiments were carried out for each sample and the results were subjected to either one-way ANOVA or Student *t*-test for comparison. A *p* value of < 0.05 was considered as statistically significant.

## 3. Results

### 3.1. HCV NS5A Induces Autophagy

**Figure 1:**
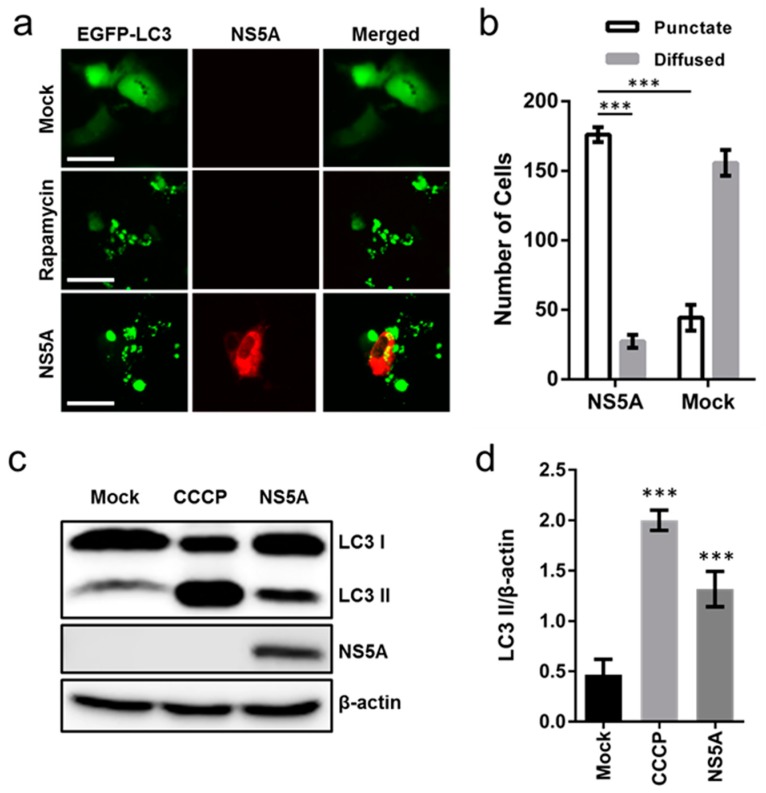
Transient transfection of NS5A increases punctate formation. (**a**) Huh-7.5 cells were co-transfected with EGFP-LC3 and NS5A or EGFP-LC3 only (mock) for 48 h after which the cells were immunostained with anti-NS5A antibody (9E10) and examined for punctate formation. Rapamycin = 100 nM; magnification = 200×; scale bar = 50 μm. (**b**) Quantification of punctate and diffused cells from a. A total of 200 cells were randomly counted and the numbers of punctate and diffused cells were determined in NS5A-transfected cells and the mock transfection control group. (**c**) Huh-7.5 cells were transfected with NS5A in 6-well plates and examined for LC3 lipidation 48 h post-transfection by extracting cell lysate for Western blotting analysis. (**d**) Densitometry of (**c**). Results are from 3 independent repeats with errors bars indicating mean ± standard deviation (SD); representative micrographs and Western blots are shown. β-actin served as a loading control for all analyses. Asterisks (*) denote statistical significance for the indicated parameters analyzed (**b**) or compared with mock (**d**): ***p < 0.001.

Although HCV has been shown to induce auto/mitophagy, the principle viral protein responsible and the underlying mechanism remain elusive. Due to the importance of HCV NS5A in the viral life cycle and its ability to modulate a plethora of cellular activities, we hypothesized that the viral phosphoprotein may play a key role in influencing host cell auto/mitophagy. For this purpose, we first investigated its ability to induce autophagy by overexpressing EGFP-LC3 with or without NS5A’s presence in Huh-7.5 cells, and monitored for LC3-associated punctate formation via fluorescence microscopy. The cytosolic LC3 (non-lipidated form; ‘LC3I’) is covalently linked to phosphatidylethanolamine (lipidated form; ‘LC3II’) upon the induction of autophagy and its redistribution to the newly formed punctate autophagosomes serves as an autophagy marker [19]. As shown in Figure 1a, in contrast to the mock-transfected group which exhibited a diffused pattern of EGFP-LC3, NS5A overexpression increased punctate formation. Similar effects were observed in Huh-7.5 cells treated with rapamycin, an mTOR inhibitor that is commonly used to induce autophagy [20]. Quantification analysis indicated a three-fold increase in punctate-forming cells when NS5A was overexpressed compared to mock transfection (Figure 1b). To further examine NS5A’s induction of autophagy, we overexpressed NS5A in Huh-7.5 cells for 48 h and performed a Western blot to monitor LC3 lipidation. As shown in Figure 1c and its associated densitometry analysis in Figure 1d, NS5A overexpression increased the lipidated form of LC3 compared to mock transfection. Treatment with CCCP, an ionophore that dissipates hydrogen gradient and commonly used to induce mitophagy and LC3 lipidation [21], also showed similar results.

### 3.2. HCV NS5A Induces Membrane Potential Loss and Mitochondrial Fragmentation

**Figure 2:**
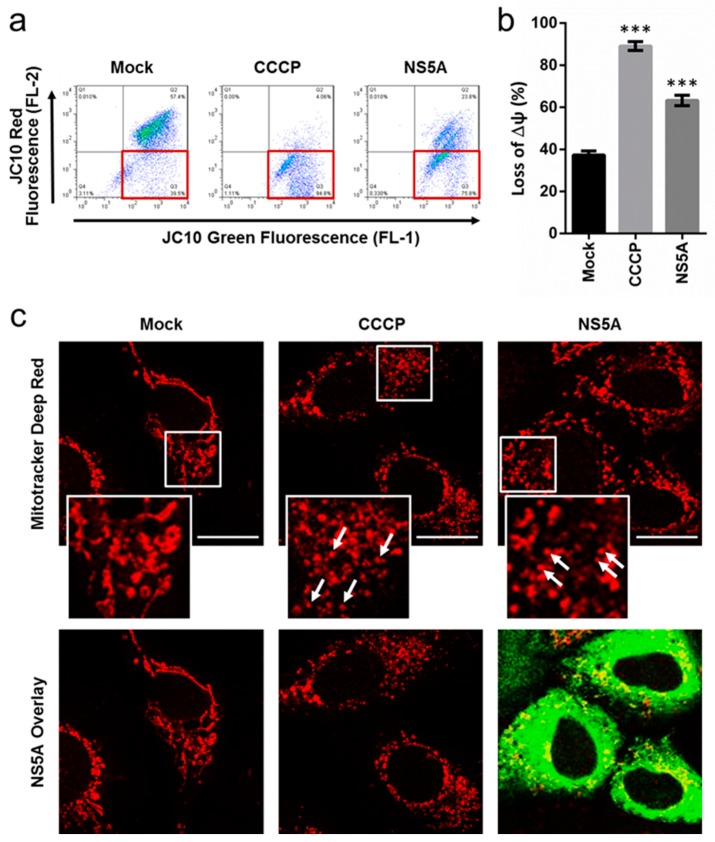
NS5A depolarizes the mitochondria and induces mitochondrial fragmentation. (**a**) Mitochondrial depolarization induced by NS5A overexpression in Huh-7.5 cells. Cells were transfected with NS5A for 48 h after which they were harvested for flow cytometry analysis using JC10 Membrane Potential Kit. Quadrant shift indicative of loss of MMP is demarcated in red. (**b**) Quantification of a. (**c**) Mitochondrial fragmentation analysis in NS5A-overexpressing Huh-7.5 cells. Huh-7.5 cells were transfected with or without NS5A on a glass slide for 3 days; CCCP treatment (10 μM) was used as positive control. The samples were stained with MitoTracker Deep Red and fixed with 4% PFA, after which the cells were stained with anti-NS5A antibodies. Magnification = 630×; scale bar = 20 μm. Demarcated areas (white squares) are shown with arrows indicating fragmented mitochondria. Results are from 3 independent experiments with errors bars indicating mean ± SD; representative flow cytometry plots and micrographs are shown. Asterisks (*) denote statistical significance compared with mock: ***p < 0.001.

Mitophagy is preceded by loss of mitochondrial membrane potential (MMP) and NS5A has been previously shown to induce calcium trafficking to the mitochondria and the production of ROS [22], which are both linked to the loss of MMP [23]. To clarify the link between HCV NS5A and mitophagy, we next examined the viral protein’s ability to trigger this mitochondria-specific event. To this end, we determined the MMP of Huh-7.5 cells transiently expressing NS5A versus the mock-transfected cells. As demonstrated in Figure 2a and its associated densitometry analysis in Figure 2b, NS5A overexpression led to a significant loss of MMP, as shown by signals accumulating in the lower right quadrant, compared to the mock control. To support this observation, we next performed confocal microscopy to examine the effect of NS5A on mitochondrial integrity. Under normal conditions, tubular and elongated mitochondria are known to resist mitophagy, whereas fragmented mitochondria due to loss of MMP allows its autophagic degradation [24]. As anticipated and depicted in Figure 2c, mock-transfected cells exhibited the tubular type mitochondria. In contrast, overexpression of NS5A induced mitochondrial fragmentation, which was also similarly observed in cells treated with CCCP. Interestingly, the NS5A staining colocalized (‘yellow spots’) with the mitochondria, suggesting a potential interaction between the viral protein and the organelle (Figure 2c). Taken together, the above results indicate that HCV NS5A disrupts mitochondrial dynamics, which may contribute to the induction of mitophagy.

### 3.3. HCV NS5A Induces Mitophagy by Triggering Parkin Translocation and LC3 Lipidation in the Mitochondria

**Figure 3:**
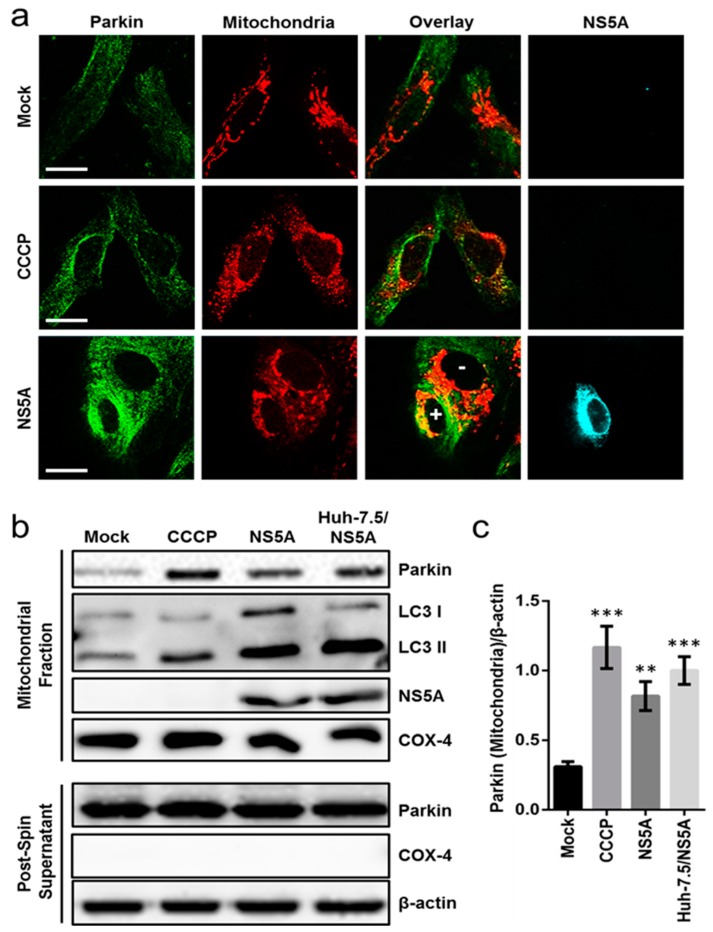
NS5A induces mitophagy. (**a**) Huh-7.5 cells were seeded on glass slides and transfected with NS5A for 3 days. The cells were subsequently pre-stained with MitoTracker Deep Red and then immunostained with anti-Parkin and anti-NS5A antibodies. Magnification = 630×; scale bar = 20 μm. The positive (+) and negative (−) symbols indicate detection of NS5A expression. (**b**) Huh-7.5 cells were seeded in a 10 cm dish and transfected with NS5A for 3 days. Cells were harvested and the mitochondrial fraction was separated using the Mitochondrial Isolation Kit for Cultured Cells, after which an immunoblot was performed for Parkin translocation and LC3 lipidation. Parkin expression in the post-spin supernatant was included for comparison. COX-4 served as mitochondrial marker. (**c**) Densitometry quantification of the mitochondrial fraction Western blot in (**b**). CCCP (10 μM) treatment on naïve Huh-7.5 cells was included as positive control. Results are from 3 independent repeats with error bars indicating mean ± SD; representative micrographs and Western blots are shown. β-actin served as a loading control for all analyses. Asterisks (*) denote statistical significance compared with mock: ** *p* < 0.01; *** *p* < 0.001.

To further examine the influence of NS5A on the induction of mitophagy, we next assessed the impact of NS5A on Parkin trafficking between the cytosol and the mitochondria via immunofluorescence analysis. Parkin translocation to the mitochondria is a distinguishing characteristic of mitophagy [25]. Huh-7.5 cells were seeded on a glass slide and transfected with or without NS5A. The cells were pre-stained with MitoTracker Deep Red and then immunostained with anti-Parkin antibody. As depicted in Figure 3a, NS5A overexpression led to the colocalization of Parkin with the mitochondria (‘yellow’) in NS5A-positive cells (marked by ‘+’ symbol), which stood in contrast to cells without NS5A expression (indicated by ‘−’ symbol) and to the mock control group. Similar results were observed in the CCCP-treatment group (Figure 3a). To validate the confocal microscopy data, we isolated the mitochondrial fraction and post-spin supernatants from NS5A-overexpressing cells and the mock-transfected control, and probed for Parkin expression by Western blot. As anticipated, Parkin translocation to the mitochondria was significantly enhanced in the mitochondrial fraction of NS5A-overexpressing cells compared to the mock control group (Figure 3b and the associated densitometry analysis in Figure 3c), corroborating the preceding results. Similar observations were noted in Huh-7.5 cells stably expressing NS5A (Huh-7.5/NS5A) (Figure 3b,c). Furthermore, in support of the Parkin translocation results, LC3 lipidation in the mitochondrial fraction (which is indicative of mitophagy induction) was also observed in the NS5A-overexpressing cells and the CCCP treatment group in contrast to the mock control (Figure 3b). Taken together, these data provide evidence that expression of NS5A, whether transient or stable, causes Parkin translocation and induces mitophagy.

### 3.4. HCV NS5A Induces Complete Auto/Mitophagy

**Figure 4:**
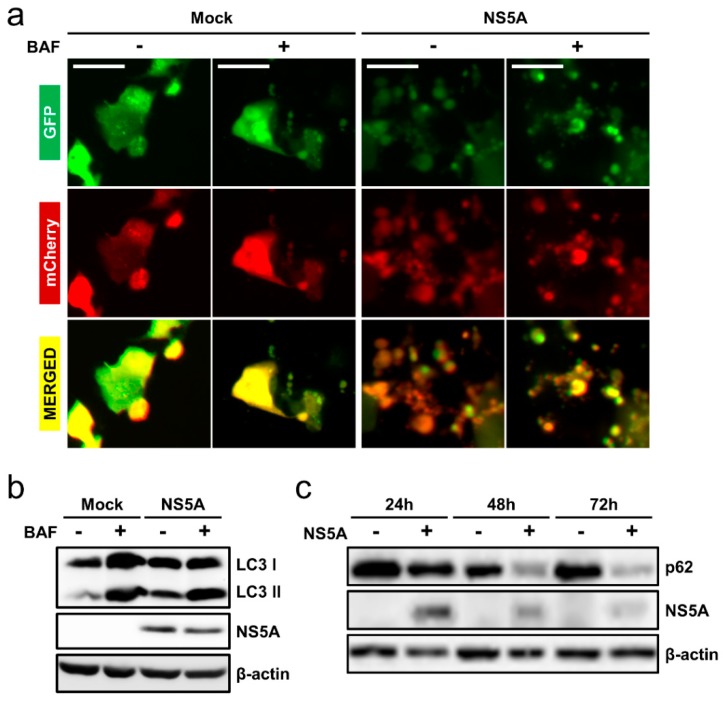
NS5A induces complete auto/mitophagy. (**a**) Huh-7.5 cells were seeded in 12-well plates and co-transfected with mCherry-EGFP-LC3 and NS5A or mCherry-EGFP-LC3 only (mock). The cells were incubated for 48 h, after which they were treated with or without 0.2 μM BAF for 12 h before fluorescence imaging. Magnification = 400×; scale bar = 20 μm. (**b**) LC3-turnover assay. Huh-7.5 cells were mock- or NS5A-transfected for 48 h and then treated with or without 0.2 μM BAF for 4 h before cell harvest for immunoblotting against LC3 and NS5A. (**c**) Analysis of p62 degradation in Huh-7.5 cells transfected with or without NS5A. The cell lysates were harvested at the indicated time-points and subsequently analyzed by Western blotting by probing with anti-p62 and anti-NS5A antibodies. β-actin served as a loading control for all analyses. Results shown are representative data from three independent experiments.

While the induction of auto/mitophagy by HCV is unquestionable, debate still exists over whether HCV infection causes complete (induction with autophagosome-lysosome fusion) or incomplete (induction without autophagosome-lysosome fusion) autophagy [26]. To examine whether HCV NS5A induces complete autophagy or autophagic flux, we used the double-tagged mCherry-EGFP-LC3. Whereas the EGFP signal is sensitive to acidic conditions and is quenched upon autophagosome-lysosome fusion, the mCherry signal is acid stable. Thus, the colocalization of the two fluorescent proteins (yellow punctate) indicates the presence of autophagosome without lysosomal fusion. On the other hand, red punctate denote autophagosome-lysosome fusion and hence completion of autophagy. Huh-7.5 cells were transfected alone with mCherry-EGFP-LC3 or co-transfected with NS5A for 48 h and then treated with the autophagosome-lysosome fusion inhibitor BAF for 12 h before microscopy. BAF is a vacuolar hydrogen ATPase inhibitor that prevents the acidification of lysosome and thus leads to accumulation of un-degraded autophagosomes (yellow punctate) when autophagy is active [27]. As shown in Figure 4a, NS5A overexpression induced autophagic flux, which is indicated by the red punctate. Moreover, BAF treatment halted autophagosome-lysosome fusion and induced the accumulation of un-degraded autophagosomes (yellow punctate) in NS5A-overexpressed cells (Figure 4a). In contrast, mock transfection (mCherry-EGFP-LC3 only) irrespective of BAF treatment did not induce significant punctate formation (Figure 4a). To further substantiate these observations, we conducted a Western blot analysis of LC3 turnover, which results in the accumulation of the lipidated LC3II in the presence of a lysosomal fusion inhibitor when autophagy is complete. Huh-7.5 cells were transfected with or without NS5A for 48 h and treated with BAF for 4 h before protein harvest. As shown in Figure 4b and in agreement with Figure 3a, BAF treatment led to the accumulation of LC3II in NS5A-overexpressed cells. To confirm the induction of autophagic flux, we performed a p62 Western blotting by harvesting Huh-7.5 cells transfected with or without NS5A at 24, 48, and 72 h post-transfection. The p62 is a protein degraded by autophagy and thus used as a marker of autophagic degradation [20]. As depicted in Figure 4c, NS5A overexpression resulted in a time-dependent degradation of p62 in contrast to the mock control group, signifying autophagy completion. The above results therefore suggest that NS5A indeed induces autophagic flux.

### 3.5. Inhibition of ROS Attenuates NS5A-Induced Auto/Mitophagy

**Figure 5:**
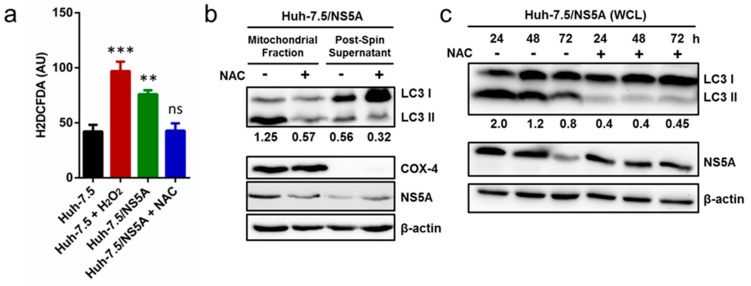
Inhibition of ROS Attenuates NS5A-Induced Auto/Mitophagy. (**a**) Huh-7.5 cells and Huh-7.5/NS5A were seeded in 6-well plates overnight followed by treatment of the Huh-7.5/NS5A cells with or without 20 mM NAC for 48 h. Subsequently, the cells were labelled with 20 μM H_2_DCFDA for 30 min and immediately analyzed by flow cytometry. Huh-7.5 cells treated with 1 mM H_2_O_2_ for 30 min served as positive control. The mean fluorescent intensity values ± SD are shown. (**b**) Huh-7.5/NS5A cells were seeded in 10 cm dishes and treated with or without 20 mM NAC for 48 h. Cells were harvested for isolation of the mitochondrial fractions and used to perform a Western blot for the indicated proteins. COX-4 served as mitochondrial marker. (**c**) Huh-7.5/NS5A cells were seeded in 6-well plates and treated with or without 20 mM NAC. The whole cell lysates (WCL) were harvested at the indicated time-points post-treatment and analyzed by Western blotting. Quantitative data and representative immunoblots are from 3 independent experiments. Numbers shown in the Western blot panels indicate the expression levels of LC3II relative to β-actin, which served as a loading control. Asterisks (*) denote statistical significance compared with mock: ** *p* < 0.01; *** *p* < 0.001; ns = not significant.

HCV NS5A has been previously reported to induce ROS production in human hepatoma cells [22,28]. On the other hand, ROS has been suggested to play an important role in the induction of autophagy [29,30]. To better clarify the underlying mechanism, we investigated the relationship between NS5A-induced mitophagy and ROS production. Naïve cells (Huh-7.5) or cells stably expressing NS5A (Huh-7.5/NS5A) were treated with or without NAC, an antioxidant that reduces oxidative stress, for three days and examined for ROS production using the indicator of oxidative stress H_2_DCFDA by flow cytometry. As depicted in Figure 5a, NS5A overexpression significantly increased ROS production as demonstrated by the augmented mean fluorescence intensity in the Huh-7.5/NS5A cells compared to the naïve Huh-7.5 control group. A similar observation was made in the H_2_O_2_ treatment group which served as a positive control. Importantly, treatment with NAC attenuated the NS5A-induced ROS production to basal level. To confirm this result, we scavenged ROS in cells stably expressing NS5A with NAC and isolated the mitochondrial fraction and post-spin supernatant to probe for LC3 lipidation by Western blot analysis. As shown in Figure 5b, in contrast to the un-treated controls (Huh-7.5/NS5A cells without NAC treatment), ROS inhibition decreased the lipidation of LC3 in the mitochondrial fraction as well as in the post-spin supernatant. Similar results were observed in the whole cell lysates of NAC-treated cells overexpressing NS5A (Figure 5c). Taken together, these results suggest ROS as an important factor in NS5A-induced auto/mitophagy.

### 3.6. HCV Core Expression Blocked Mitophagy Induction in Cells Harboring HCV Replicons or Overexpressing NS5A

**Figure 6:**
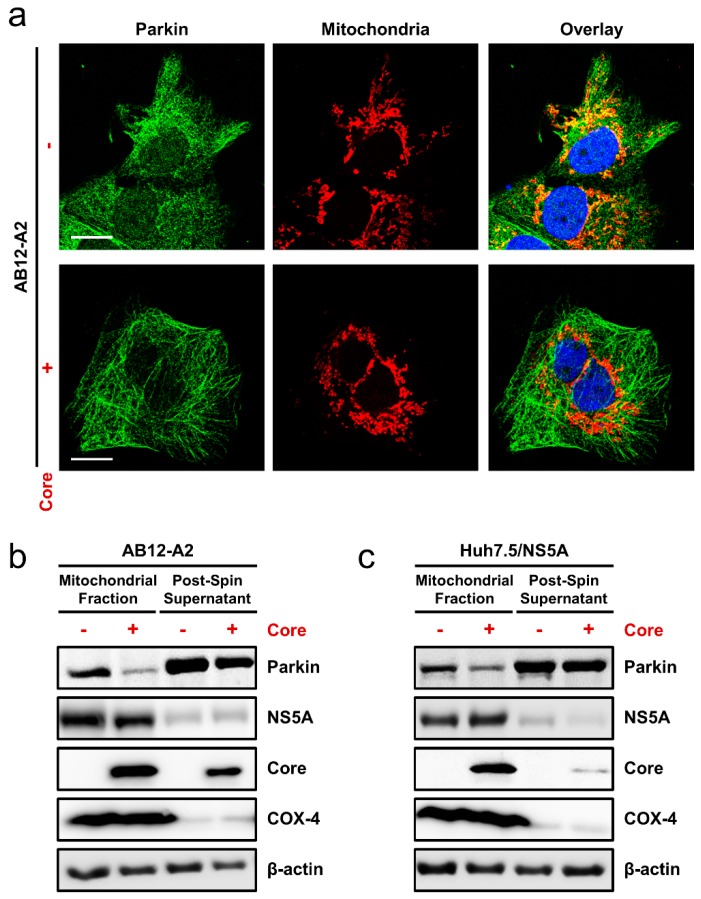
HCV Core Expression Blocks Mitophagy. (**a**) HCV subgenomic replicon AB12-A2 cells were seeded in 24-well plates and transfected with or without HCV core for 48 h for immunofluorescence analysis against Parkin and the mitochondria followed by confocal microscopy. Magnification = 630×; scale bar = 35 μm. (**b**,**c**) AB12-A2 replicons and Huh-7.5/NS5A cells were seeded in 10 cm dishes and transfected with or without HCV for 48 h. The cells were then fractionated using the mitochondrial isolation kit and subjected to Western blot analysis against the indicated antibodies. Representative immunoblots from 3 independent experiments are shown.

To further substantiate our findings on NS5A as an important regulator of the HCV-induced auto/mitophagy, we next employed HCV subgenomic replicon cells. This model harbors self-replicating mini HCV genomes generally encoding only the nonstructural proteins NS3-NS5B and recapitulates the intracellular steps of HCV replication [31]. Using fluorescence microscopy and Western blotting, we first confirmed that the Huh-7-based HCV subgenomic replicon AB12-A2 cells [17] express the NS5A protein and induce autophagic flux (Figure S1). Next, we sought to investigate whether the HCV subgenomic replicon cells induce mitophagy and whether HCV core expression, which has been demonstrated to block Parkin translocation to the mitochondria and hence mitophagy [32], could impede the AB12-A2-induced mitophagy. AB12-A2 replicon cells were seeded and transfected with or without HCV core for immunofluorescence analysis against Parkin and mitochondria. As shown in Figure 6a upper panel, significant Parkin translocation was observed with mitochondrial colocalization (yellow spots) in the AB12-A2 replicon cells, suggesting efficient induction of mitophagy by the replicon cells. In contrast, expression of HCV core strongly inhibited the replicon cells-induced mitophagy, indicated by the substantial loss of colocalization (red spots) between Parkin and the mitochondria in HCV core-expressing cells compared to the AB12-A2 replicon cells alone (Figure 6a, lower panel). To validate the above results, we seeded the AB12-A2 cells and transfected with or without HCV core for Western blot analysis against Parkin. Similar to the immunofluorescence assay, HCV core expression robustly attenuated the replicon cell-induced enrichment of Parkin in the mitochondria (Figure 6b). Similar results were also observed when HCV core was introduced in cells solely expressing NS5A (Figure 6c). Taken together, the above results demonstrated that HCV core expression impeded the mitophagy induction in cells harboring the HCV replicating subgenomes or expressing NS5A thereby pinpointing the viral phosphoprotein as an important regulator of cellular auto/mitophagy.

## 4. Discussion

The mitochondria are dynamic organelles whose quality control is crucial for cell viability and the maintenance of the overall cellular homeostasis. HCV infection and the expression of its proteins have been reported to induce mitochondrial injury [33]. Accordingly, biopsies from HCV-infected hepatocytes often manifest signs of swollen and ruptured mitochondria [34]. Since the accumulation of damaged mitochondria is detrimental to cell survival [35], quality control of the injured mitochondria via mitophagy is crucial for the survival of the HCV-infected cells [36]. Interestingly, HCV NS5A has previously been reported to inhibit apoptosis and promote cell survival in human hepatoma cell lines [10,37,38]. Our finding that NS5A can specifically induce the recycling of damaged mitochondria could additionally explain how this viral protein promotes cell survival, which in turn has implications in favoring viral persistence. Our results therefore add to NS5A’s pleotropic functions in regulating cellular pathways and highlight the role of NS5A in promoting the HCV pathogenesis.

HCV is adept at causing chronic infection partly because of its numerous immune escape strategies, such as the cleavage of the mitochondrial antiviral signaling protein (MAVS) by the NS3/4A protease to inhibit the downstream IFN signaling [39] and the binding of NS5A to the protein translation inhibitor, the double-stranded RNA-dependent protein kinase PKR [40]. Recently, HCV-induced mitophagy has been suggested to contribute to the virus’ innate immune evasion, which could potentially be mediated by the induction of mitochondrial fragmentation [36]. Herein, we demonstrate that NS5A expression alone, in addition to inducing MMP loss, also induces mitochondrial fragmentation, which we speculate could weaken the innate immune signaling by triggering the recycling of the mitochondria through mitophagy. In support of this hypothesis, loss of MMP and mitochondrial fragmentation have been suggested to decrease RIG-I-like receptor (RLR) signaling [41,42], which is known to mediate the antiviral recognition of HCV RNA [43].

HCV infection is known to induce autophagy [44,45], but whether the virus causes complete or incomplete autophagy remains debatable. While Sir et al. suggest the induction of incomplete autophagy by HCV [46], a growing body of literature argues that HCV induces complete autophagy [47,48,49]. In agreement with the latter studies supporting the notion of autophagic flux, we demonstrated that HCV NS5A overexpression causes a time-dependent degradation of p62 (Figure 4c) and an accumulation of un-degraded autophagosomes in our LC3-turnover assay (Figure 4b), both of which correlate with complete auto/mitophagy. Similar results were observed in the AB12-A2 HCV subgenomic replicon cells (Figure S1). Interestingly, we also observed that NS5A colocalizes with both LC3 (Figure 1a) and the mitochondria (Figure 2c), and was highly enriched in the mitochondrial fraction (Figure 3b and Figure 6b,c). The fact that NS5A colocalizes with the mitochondria and was enriched in the mitochondrial fraction suggests that the viral protein could possibly interact with the organelle. Further studies are needed to clarify how exactly NS5A triggers and regulates the auto/mitophagy pathway in the mitochondrial compartment, and the critical region(s) of NS5A responsible for these effects.

HCV infection is highly associated with oxidative stress that is suggested to contribute to the development of HCC [50]. Interestingly, HCV core and NS5A are the major proteins implicated in HCV-induced ROS production [51]. Here, we also observed an increase in ROS production in NS5A-overexpressing cells (Figure 5a) as well as in the AB12-A2 replicon cells (Figure S2). Importantly, we discovered that the NS5A-mediated ROS production is crucial to the viral phosphoprotein’s induction of auto/mitophagy, since inhibition of ROS attenuated the NS5A-induced auto/mitophagy with significant decrease in mitochondrial and non-mitochondrial LC3II levels (Figure 5c). How ROS triggers the NS5A-induced auto/mitophagy is not clear. Recently, ROS was reported to contribute to the hepatitis B virus X protein (HBx)-induced autophagy via regulating Beclin1/Bcl-2 interaction [52]. Specifically, HBx-induced ROS led to the phosphorylation of Bcl-2, thus freeing Beclin1 from its association to induce autophagy. Beclin 1 plays a crucial role in the induction of autophagy by forming a complex with VPS34 [53] and its interaction with the anti-apoptotic protein Bcl-2 downregulates its own expression, thereby inhibiting autophagy [54]. Interestingly, we also observed an increase in Beclin 1 protein expression in NS5A-overexpressing cells (data not shown), suggesting a potential regulation at the Beclin 1 level. Further studies will be needed to clarify whether a link exists between the NS5A-induced ROS production and the dissociation of the Bcl-2/Beclin 1 complex to promote auto/mitophagy.

In contrast to the report by Kim et al. that HCV induces mitophagy [16], a recent report indicated that HCV infection inhibits mitophagy in an HCV core-dependent manner. Specifically, HCV core was demonstrated to interact with Parkin, blocking its translocation to the mitochondria, and consequently inhibiting mitophagy [32]. Herein, we also demonstrated that HCV core expression blocked both the AB12-A2 replicon cells and NS5A-induced mitophagy (Figure 6), supporting the idea that the core protein inhibits mitophagy as well as pinpointing NS5A as a key regulator of the HCV-induced mitophagy. Interestingly, we observed that despite the suppression of mitophagy by HCV core, the expression of the viral protein appears to augment the AB12-A2 replicon-induced autophagy (data not shown). Considering the delayed expression of HCV core (partly due to the rapid turnover of the protein at early time-points) relative to the other HCV proteins such as NS3 and NS5A [55], it is tempting to speculate that the spatiotemporal regulation of HCV core and NS5A protein expressions could regulate the virus-induced mitophagy. Interestingly, the transient interaction of HCV core and NS5A was reported to be critical for the production of infectious viral particles [56]. Whether this interaction between the two most promiscuous HCV proteins contributes to the regulation of mitophagy during productive HCV infection is a subject of intense interest. It is possible that HCV temporally regulates mitophagy depending on the expression level of the core protein. More in-depth research is needed to verify this hypothesis and clarify the significance of this regulation in the HCV life cycle. In summary, we have demonstrated for the first time that in addition to triggering autophagy, the HCV phosphoprotein NS5A is sufficient on its own to induce alterations in the mitochondrial dynamics that lead to the induction of mitophagy. This adds to a strong body of evidence that implicates NS5A as an important regulator of the host cell during the HCV life cycle [57]. Understanding the disruption of mitochondrial dynamics by NS5A and its relationship to mitophagy may enhance our understanding of the hepatitis C pathogenesis and identify novel targets for therapeutic intervention.

## Supplementary Materials

The following are available online at https://www.mdpi.com/2073-4409/8/4/290/s1, Figures S1 and S2.
