# Supplementary material for: Hepatitis C Virus Non-Structural Protein 5A (NS5A) Disrupts Mitochondrial Dynamics and Induces Mitophagy
Source: Cells. 2019 Mar 29;8(4):290. doi: 10.3390/cells8040290 (PMC6523690; doi:10.3390/cells8040290)
Supplement: Supplementary file 1 [file cells-08-00290-s001.pdf]

Supplementary Material

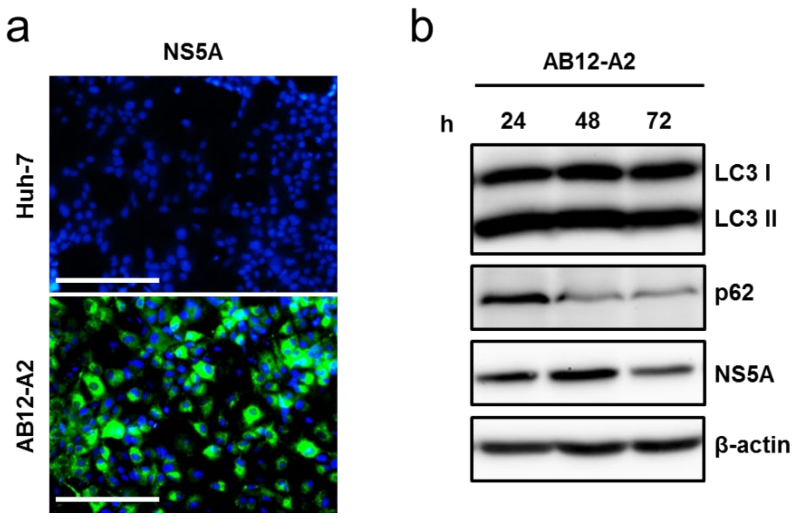

Figure S1. AB12-A2 cells express NS5A and induce autophagic flux. (a) HCV subgenomic replicon AB12-A2 cells were seeded in 24-well plates and stained with anti-NS5A antibody. The parental naïve Huh-7 cells were included as control and Hoechst stain was used to demarcate the nuclei. Magnification = 200X; scale bar = 200 μm. (b) AB12-A2 replicon cells were seeded in 6-well plates for the indicated time-points. The lysates were subsequently harvested and subjected to Western blot analysis for the indicated proteins.

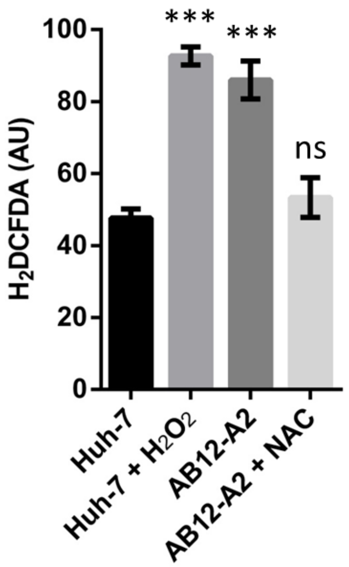

Figure S2. AB12-A2 cells induce ROS production. Huh-7 and the AB12-A2 replicon cells were seeded in 6-well plates followed by H_2DCFDA staining and flow cytometry analysis for ROS production with procedures and treatments carried out as in Fig. 5a. NAC = 20 mM; H_2O_2 = 1 mM. Asterisks (*) denote statistical significance compared with control (Huh-7 only): ***$p < 0.001$; ns = not significant.
